# Looking back at prospective modeling of outbreak response strategies for managing global type 2 oral poliovirus vaccine (OPV2) cessation

**DOI:** 10.3389/fpubh.2023.1098419

**Published:** 2023-03-24

**Authors:** Kimberly M. Thompson, Dominika A. Kalkowska, Kamran Badizadegan

**Affiliations:** Kid Risk, Inc., Orlando, FL, United States

**Keywords:** polio, eradication, oral poliovirus vaccine, dynamic modeling, immunization, outbreak response, cessation

## Abstract

**Introduction:**

Detection of poliovirus transmission and ongoing oral poliovirus vaccine (OPV) use continue to delay poliomyelitis eradication. In 2016, the Global Polio Eradication Initiative (GPEI) coordinated global cessation of type 2 OPV (OPV2) for preventive immunization and limited its use to emergency outbreak response. In 2019, GPEI partners requested restart of some Sabin OPV2 production and also accelerated the development of a genetically modified novel OPV2 vaccine (nOPV2) that promised greater genetic stability than monovalent Sabin OPV2 (mOPV2).

**Methods:**

We reviewed integrated risk, economic, and global poliovirus transmission modeling performed before OPV2 cessation, which recommended multiple risk management strategies to increase the chances of successfully ending all transmission of type 2 live polioviruses. Following OPV2 cessation, strategies implemented by countries and the GPEI deviated from model recommended risk management strategies. Complementing other modeling that explores prospective outbreak response options for improving outcomes for the current polio endgame trajectory, in this study we roll back the clock to 2017 and explore counterfactual trajectories that the polio endgame could have followed if GPEI had: (1) managed risks differently after OPV2 cessation and/or (2) developed nOPV2 before and used it exclusively for outbreak response after OPV2 cessation.

**Results:**

The implementation of the 2016 model-based recommended outbreak response strategies could have ended (and could still substantially improve the probability of ending) type 2 poliovirus transmission. Outbreak response performance observed since 2016 would not have been expected to achieve OPV2 cessation with high confidence, even with the availability of nOPV2 prior to the 2016 OPV2 cessation.

**Discussion:**

As implemented, the 2016 OPV2 cessation failed to stop type 2 transmission. While nOPV2 offers benefits of lower risk of seeding additional outbreaks, its reduced secondary spread relative to mOPV2 may imply relatively higher coverage needed for nOPV2 than mOPV2 to stop outbreaks.

## 1. Introduction

Look back analyses can provide feedback and insights that improve prospective modeling activities by comparing model predictions to observed outcomes and identifying model assumptions that explain any differences (1, 2). As analysts with experience prospectively modeling the polio endgame for over 2 decades, we rely on this type of feedback (3, 4). Although globally-coordinated efforts in 2016 sought to permanently end all cases of poliomyelitis (polio) caused by type 2 polioviruses, as of 2023, these cases currently dominate the annual reported incidence (5).

Before 2000, the partners of the Global Polio Eradication Initiative (GPEI) recognized that achieving the goal of ending poliomyelitis (6) would require stopping all use of oral poliovirus vaccines (OPVs) following global certification of the eradication of indigenous transmission of wild polioviruses (WPV) (7). Notably, after successful WPV eradication, continued use of OPV with insufficient coverage can create conditions in which OPV-derived viruses evolve to lose their attenuating mutations as they transmit, leading to outbreaks of circulating vaccine-derived polioviruses (cVDPVs) (8, 9). With trivalent OPV (tOPV, containing OPV for all three types of polioviruses) as the only licensed OPV formulation at the time used for routine immunization (RI) and supplementary immunization activities (SIAs), these early discussions considered several options (7). The options included stopping all tOPV use after certification of all WPVs (i.e., OPV cessation) or phasing OPV cessation for each of the 3 poliovirus types [e.g., starting with cessation of OPV type 2, referred to initially as “II-less” (7) or “2-less” (10) OPV].

In 2008, global health leaders resolved “to set, if and when appropriate, a date for the eventual cessation of use of oral poliomyelitis vaccine use in routine immunization….” (11) Not surprisingly, the risks of type 2 cVDPVs (cVDPV2s) increased due to the use of various OPV vaccine formulations that did not include type 2 OPV (OPV2) for SIAs [i.e., monovalent OPV (mOPV) for type 1 (mOPV1) or type 3 (mOPV3), bivalent OPV (bOPV, containing types 1 and 3)] (9, 12, 13). With reported polio cases for 2001–2012 hovering between 250 and 2,050 per year (14), delays in achieving the eradication of type 1 WPV (WPV1), and increasing cVDPV2 incidence, the GPEI 2013-2018 Strategic Plan included phased OPV cessation starting with OPV2 in 2016 (15).

Preparation for OPV2 cessation considered the insights from integrated risk, economic, and global poliovirus transmission modeling (3). A 2012 study explored OPV cessation options and identified five prerequisites for success: (1) an appropriate vaccine stockpile for outbreak response, (2) emergency response plans and standard operating procedures (SOPs), (3) high type 2 population immunity in all countries, (4) ending all of the existing transmission of cVDPV2s prior to OPV cessation, and (5) developing plans for (i) managing the risks associated with the very small number of immunodeficient VDPV excreters, (ii) containing type 2 live polioviruses, (iii) maintaining high-quality surveillance, and (iv) ensuring sufficient quantities of the polio vaccines that countries would continue to use for RI (12). Emphasizing the importance of increasing population immunity prior to OPV cessation (16), prior work particularly called out Nigeria, Pakistan, and Afghanistan as high risk geographies for cVDPV2 outbreaks (12). Other studies further supported the recommended prerequisites by explaining the need for intensifying type 2 population immunity prior to cessation (17) using tOPV in SIAs (13). The recommendations from modeling did not include adding inactivated poliovirus vaccine (IPV) to RI as a prerequisite to OPV2 cessation given its limited role in stopping and preventing poliovirus transmission (18). Modeling of IPV use in the polio endgame recognized its potential role as providing insurance against paralysis for IPV recipients in the event that OPV cessation failed, but also highlighted that the use of IPV could delay the detection of polio outbreaks by preventing cases (12, 18).

After consideration of the insights from the modeling and the associated recommended management strategies to increase the chances of successfully ending all transmission of type 2 live polioviruses, the GPEI decided on its readiness criteria (19) and process for OPV2 cessation (20). The GPEI included IPV introduction as a prerequisite to OPV2 cessation and aggressively pursued the introduction of at least one dose of IPV into all national immunization program schedules prior to or shortly after OPV2 cessation (20). Subsequent global modeling that included this planned IPV introduction developed recommendations for outbreak response SOPs and the vaccine stockpile (21), highlighting polio endgame vaccine needs (22), and demonstrated the combined importance of managing all risks well (23). Global modeling estimated an ~94% chance that following the recommended outbreak response strategies would lead to successful OPV cessation (23). Other modeling studies also identified risk management opportunities to potentially further increase the chances of success or develop contingencies in the event of failure by exploring the potential development of polio antiviral drugs (24) and/or new polio vaccines (25, 26).

The epidemiological conditions at the time of OPV2 cessation, actual experience with outbreak response, and development of nOPV2 as an alternative vaccine for outbreak response raise questions about how the integrated modeling in 2016 would have looked with the different initial conditions, options, and actions. Here, we roll back the clock to 2017 and explore counterfactual prospective trajectories that the polio endgame could have followed if the countries and the GPEI had managed outbreaks differently after OPV2 cessation. We start by identifying the initial conditions that existed at the time of OPV2 cessation [instead of more optimistic conditions used in pre-OPV2 cessation modeling (21, 23)] and risk inputs consistent with observations since 2016 (27, 28). In addition, the accelerated development, emergency use listing, and initial use of nOPV2 (29) has raised questions about whether outcomes might have differed if nOPV2 had been available for oSIAs starting in 2017. Insights from this analysis may help guide prospective decisions related to bOPV cessation and management of the global poliovirus transmission risks and contribute to documentation of lessons learned from OPV2 cessation.

## 2. Review of published post-OPV2 cessation modeling and epidemiological experience

Global certification of eradication of transmission of indigenous type 2 wild polioviruses in 2015 (30), set the stage for globally-coordinated OPV2 cessation in 2016 (20). After May 2016, OPV2 use was restricted to emergency outbreak response to cVDPV2 outbreaks using type 2 monovalent Sabin OPV (mOPV2) provided from a global stockpile controlled by the Director General of the World Health Organization (WHO), which the GPEI created as a prerequisite to OPV2 cessation (12, 21, 31).

After mid-2016, many studies reported on the status of OPV2 cessation to evaluate the quality of the prospective assumptions made about risks prior to OPV2 cessation (23) and highlighted issues with the risk management activities as implemented (32–36). This included several early studies that found that most countries successfully ended transmission of type 2 live polioviruses, but also identified issues with insufficient tOPV intensification prior to OPV2 cessation in some countries (e.g., Nigeria, Pakistan), which implied higher global risks of continued type 2 live poliovirus transmission at the start of OPV2 cessation than recommended by earlier modeling (32–34). These studies also emphasized an increased risk of needing to restart OPV broadly in RI if mOPV2 was not used according to the outbreak response SOPs, outbreaks occurred in very high transmission settings, and/or countries used IPV for outbreak response instead of mOPV2 (32, 33). A 2017 study highlighted that without improved access to under-vaccinated populations, neither WPV1 nor cVDPV2 transmission would likely die out (34). Following the observation of some use of IPV for outbreak response instead of the recommended mOPV2, another study demonstrated the lack of IPV effectiveness and cost-effectiveness when used for outbreak response (37). That study also highlighted: (i) mixed messages that implied or promised IPV benefits not consistent with available evidence and experience (or model expectations), (ii) the lack of urgency with respect to responding to cVDPV2 outbreaks, and (iii) observations of hesitancy with respect to using mOPV2 for outbreak response (37). As time since OPV2 cessation passed, integrated modeling increasingly demonstrated that the GPEI was not on track for successful OPV2 cessation, and emphasized the importance of ensuring the availability of sufficient numbers of filled mOPV2 doses in the global stockpile to support rapid outbreak response (35). Modeling of Pakistan and Afghanistan further suggested that the polio endgame was not on a path to zero for WPV1 in endemic countries, and anticipated the need to manage co-circulating transmission of both types 1 and 2 in these countries, while highlighting that tOPV would be a better option for outbreak response than mOPV2, if available (36).

By 2019, our integrated modeling studies began to use updated assumptions about prospective risks that reflected observed experience since 2016. These included updated estimates of potential reintroduction of transmission from individuals with primary immunodeficiencies infected with polio (i.e., immunodeficiency associated VDPVs or iVDPVs) (38, 39), containment risks (40), and logistics related to potentially restarting OPV2 use in RI (41). A reflection on the risk management strategies recommended by modeling prior to OPV2 cessation that the GPEI and countries did not implement as recommended by pre-OPV2 cessation modeling provided an overall summary of model assumptions that we needed to change to correspond with national, regional, and GPEI policies and practices (4). Subsequent modeling showed the combined impact of the updated assumptions on the polio endgame trajectory (27), as well as the consequences of updated OPV cessation risks (28) after the emergences of type 2 transmission in 2019 in Angola and Pakistan from unknown sources (42). Changing the characteristics of outbreak response SIAs (oSIAs) from those recommended prior to OPV2 cessation (21, 23) to those that actually occurred (“current”) after OPV2 cessation (Table 1) represented a key component of this updated modeling (27).

**Table 1:** Characteristics of outbreak response supplemental immunization activities (oSIAs) recommended prior to OPV2 cessation (21, 23) and currently in use after OPV2 cessation (27).

	**Recommended oSIA (21, 23)**	**Current oSIA (27)**
Vaccine choice and stockpile	mOPV2 stockpile designed for mOPV2 use up to 5 years post-OPV2 cessation, then anticipated shift to IPV	period of allowed mOPV2 use extended up to 8 years after OPV2 cessation (through 2023) (43), then extended indefinitely with anticipated use of nOPV2 (44, 45)
Scope	respond in all subpopulations in the blocks with R_0_ ≥ 10, otherwise just in outbreak subpopulation	respond in outbreak subpopulation and 4 worst-performing neighboring subpopulations within the same block when R_0_ ≥ 10, otherwise just in outbreak subpopulation
Days to first round	45 for first detection in block, 30 for other subpopulations in block	45 for first detection in block, 30 for other subpopulations in block
Size	4 rounds in blocks with R_0_ < 12, 6 rounds in blocks with R_0_ ≥ 12, all rounds 30 days apart	2 rounds separated by 30 days with 2 additional rounds after detection of breakthrough transmission
Intensity	true coverage 80%, repeatedly missed probability 70%	same as subpopulation-specific pSIAs, which ranges from true coverage 15%, repeatedly missed probability 95% to true coverage 80%, repeatedly missed probability 70% [see details in Kalkowska et al. (27)]
Target	greater of < 5 years old *or* all cohorts born since OPV cessation (whichever is larger)	< 5 years old

With anticipation that nOPV2 would replace mOPV2 by July 2021 (44), integrated modeling explored how using nOPV2 instead of mOPV2 might change the expected endgame trajectory for type 2 poliovirus transmission (46). After the COVID-19 pandemic disrupted global activities, further modeling characterized the impacts of these disruptions on the polio endgame trajectory and the expected impacts of using of nOPV2 for outbreak response instead of or in addition to mOPV2 (47–49). Although the use of nOPV2 promises greater genetic stability (50), which means lower risks of evolving to cause new outbreaks while preserving some mucosal immunological benefits induced by OPV, all of these studies highlighted the need for improved oSIA performance (47–49). These studies repeated the message that vaccine failure was not the problem, and that OPV cessation and polio eradication remain off track due to the failure to vaccinate (4, 34).

## 3. Materials and methods

For this analysis, we apply our integrated risk, economic, and dynamic disease transmission model used extensively over the past two decades to support the global polio eradication efforts (3). We focus on looking back at our modeling studies, while noting that other modeling teams provided independent analyses [reviewed elsewhere (3)] and may want to similarly perform independent look back analyses.

Most recent updates and applications of our integrated model consider prospective options for improving outcomes after responding to outbreaks for the current polio endgame trajectory for 2022–2026 (47–49). Briefly, the global model divides the world into 72 blocks according to World Bank Income Level (low-income, LI; lower middle-income, LMI; upper middle-income, UMI; high-income, HI) and current vaccine use in RI (OPV+IPV, IPV/OPV, IPV-only). The model subdivides each block into 10 subpopulations consisting of ~10.7 million total population in 2019 summing up to 7.7 billion of global population at that time. Further, the model divides each subpopulation into 7 age groups. The age distributions within the subpopulations vary consistently with variability in the global population, and on average a subpopulation includes ~1 million children < 5 years, 2 million < 10 years, and 3 million < 15 years of age. Mixing within blocks occurs homogenously in space and heterogeneously by age, while mixing between blocks occurs according to nine varying preferential mixing areas of different size, which in abstract represent larger geographical regions (e.g., continents or large countries like India and China). For each of the 720 subpopulations, the deterministic differential equation-based poliovirus transmission model uses 8 immunity states to simulate the different types and levels of immunity, induced by IPV and/or OPV (or another live poliovirus (LPV) infection) for each type of poliovirus separately, with a 5-stage waning process, considering both fecal-oral and oropharyngeal transmission, and including a 6-stage infection process (2 latent and 4 infectious stages) and a 20-stage OPV evolution process (27, 51). We characterize stochastic aspects of the poliovirus transmission by modeling random interactions and the possibility of spreading an infection between people from different geographical locations, as well as by using the probabilities of reintroduction risks associated with insufficient removal of a type of OPV from the supply chain after its globally-coordinated cessation, iVDPV excreters, and/or breeches in containment (23, 27).

Applying the integrated model with assumptions that reflect actual choices and performance, we previously modeled a deterministic run-up until December 31, 2021 and ran 100 prospective stochastic iterations for the period of January 1, 2022 to December 31, 2026, which we refer to as the current reference case (RC) for the trajectory of the GPEI (49). The current RC, as observed after OPV2 cessation, uses inputs for RI and SIAs as they occurred through December 31, 2021 (49) and assumes that prospective preventive SIAs (pSIAs) occur based on recent and current GPEI strategic plans (43, 45). With respect to the oSIAs, the RC assumes characteristics for prospective oSIAs consistent with recent performance (Table 1, rightmost column) (27). Specifically, prospective oSIAs in the current RC target children < 5 years of age, start 45 days after detection, and include 2 rounds of mOPV2 separated by 30 days, and perform 2 additional rounds after detection of breakthrough transmission (27, 49). The scope of the modeled prospective oSIAs includes the outbreak subpopulation alone for subpopulations with a type 1 wild poliovirus (WPV1) basic reproduction number (R_0_) < 10 (most subpopulations), or the outbreak subpopulation and its four worst-performing neighbor subpopulations within the same block when WPV1 R_0_ ≥ 10 (49). We characterize the performance of oSIAs using true coverage and repeatedly missed probability (*P*_*RM*_) inputs, the latter of which accounts for missing the same children between successive rounds. Consistent with observed performance, the oSIA intensity in the model varies for different subpopulations, ranging from 15% true coverage and 95% *P*_*RM*_ to 80% true coverage and 70% *P*_*RM*_ (27, 28, 46–49, 52). The RC uses mOPV2 as the primary vaccine for type 2 oSIAs for the entire time horizon as a modeling benchmark for comparisons (49).

As a complement to recent modeling that focused on the expected polio endgame trajectory as of early 2022 (46, 49), in this study, we look back and ask: Where could we have been and where would we be heading now if we had made different choices since 2016? As part of this, we answer the question: What if type 2 oSIAs after OPV2 cessation followed the recommendations from our pre-OPV2 cessation modeling (21, 23)? However, unlike the pre-OPV2 cessation modeling that assumed better tOPV intensification prior to OPV2 cessation than actually occurred (21), for this analysis, we start with the conditions that existed at the time of OPV2 cessation in 2016 and we use risk inputs consistent with observations since 2016 (27, 28). For this analysis, we show the current RC for context, but we focus on alternative scenarios that represent counterfactuals for the time horizon of 2016–2026. Table 2 provides an overview of the conditions and assumptions for prospective modeling performed pre-OPV2 cessation (before 2016, second column), context about the actual experience observed since OPV2 cessation (third column), and for current post-OPV2 cessation modeling in the RC (fourth column). The last column summarizes the conditions and assumptions for the prospective modeling performed for this look back analysis (fifth column), with the bottom three rows showing the different permutations of assumptions for the counterfactual analyses.

**Table 2:** Overview of conditions and assumptions for prospective modeling studies performed pre- and post-OPV2 cessation, actual observations that motivated post-OPV2 cessation modeling changes, and alternative scenarios considered in this look back analysis.

←**2016**		**2017**→		
	Pre-OPV2 cessation	Context about actual experience	Post-OPV2 cessation reference case (RC)	Look back modeling (counterfactual analysis)
Population immunity initial conditions	tOPV intensification to achieve high population immunity and end persistent cVDPV2s	Insufficient tOPV intensification in some countries (e.g., Nigeria, Pakistan)	Deterministic modeling of actual RI and SIAs through 2021 with prospective assumptions from 2022 on	Deterministic modeling of actual RI and SIAs through 2016 with prospective assumptions from 2017 on
Reintroduction risks	Based on prior experience and uncertainty estimates for: 1) Incomplete removal of OPV2 from supply chain for 1 year after cessation 2) Unintentional and intentional releases from manufacturers and other 3)Higher-bound iVDPV excreter risk	Updated based on observations and evidence obtained through end 2021 1) Higher and longer reintroduction risks due to incomplete removal of OPV2-containing vaccines from supply chains after cessation and/or use in oSIAs 2) Updated characterization of unintentional and intentional releases depending on type and location of manufacturers and specific types of other facilities 3) Lower iVDPV excreter risks due to limited observations after OPV2 cessation		
OPV2 restart in RI	OPV2 restart in RI if cumulative cases after cessation exceed threshold	No OPV2 restart		
oSIA vaccine choices	1) mOPV2 use from stockpile (designed to last for up to 5 years post-cessation) 2) Shift to IPV after 5 years	1) mOPV2 use from initial stockpile and new bulk production 2) No switch to IPV and continued OPV2 use (shift from mOPV2 to nOPV2)	1) Model actual vaccines used for SIAs through 2021 2) From 2022 on, model 3 vaccine options for SIAs: i) mOPV2, ii) nOPV2 no reversion, OR iii) nOPV2 some reversion	1) Model actual vaccines used for SIAs through 2016 2) From 2017 on, model 3 vaccine options for SIAs: i) mOPV2, ii) nOPV2 no reversion, OR iii) nOPV2 some reversion
oSIA performance (Table 1)	“Recommended oSIA”	“Current oSIA”		i) “Current oSIA” OR ii) “Recommended oSIA”
IPV in RI in all countries required prior to OPV2-cessation (prerequisite)	No IPV prerequisite recommended by modeling, however, due to GPEI readiness criteria, modeling included at least 1 dose of IPV in RI in all countries introduced by 2017	Delayed IPV introduction in some countries	1) Model actual use of IPV through 2021 2) From 2022 on, include 1 dose of IPV in RI with projected coverage	1) Model actual use of IPV through 2016 2) From 2017 on: i) no IPV prerequisite, OR ii) include 1 dose of IPV in RI with projected coverage

For the alternative scenarios, we use the same deterministic run-up as the RC through December 31, 2016 (shortly after OPV2 cessation) and perform 100 stochastic iterations for the period of January 1, 2017 to December 31, 2026 to investigate alternative stochastic pathways that could have occurred during the post-OPV2 cessation period. We assume the same RI and pSIAs as the RC for the entire time horizon, including IPV as introduced and now used in all countries (i.e., *IPV in all RI*). However, we consider one set of scenarios that do not include the introduction of IPV into RI in the LI and LMI blocks as a prerequisite to OPV2 cessation (i.e., *no IPV prerequisite*), for which we assume no IPV use in these blocks during the entire model time horizon. Building on the observation of numerous prior studies that raised issues about the performance of oSIAs since OPV2 cessation (27, 28, 32–35, 38, 46, 48, 49), we consider 3 different oSIA vaccine choice options (49): (i) mOPV2 for the entire time horizon as assumed for the RC (i.e., *mOPV2*), (ii) nOPV2 with the same effectiveness and rate of vaccine-associated paralytic polio (VAPP) as mOPV2, but no reversion to wild-type poliovirus phenotype despite transmissibility (i.e., *nOPV2 no reversion*), or (iii) nOPV2 of the same effectiveness and VAPP rate as mOPV2, but with some reversion to wild-type poliovirus phenotype occurring at a slower rate relative to mOPV2 (i.e., *nOPV2 some reversion*) (49). For all three vaccine choices, we consider two options of oSIA characteristics: (i) RC oSIA characteristics (i.e., *current*) and (ii) oSIA characteristics recommended by modeling prior to OPV2 cessation (21) (i.e., *recommended*) (see Table 1). Specifically, prior to OPV2 cessation, the recommended model assumptions for prospective oSIAs included: targeting the larger of either children < 5 years of age or all cohorts born since OPV2 cessation, start 45 days after detection, and used 4 rounds of mOPV2 (or 6 rounds in very high transmission blocks with WPV1 R_0_ ≥ 12) separated by 30 days followed by additional rounds after breakthrough transmission for the first 5 years after OPV2 cessation (or IPV if outbreaks occurred after that time) (21, 23, 35). The scope of the recommended prospective oSIAs includes the outbreak subpopulation alone when the WPV1 R_0_ < 10 or the entire block when WPV1 R_0_ ≥ 10, and immunization intensity that achieved 80% true coverage and 70% repeatedly missed probability in all outbreak areas (21, 23).

The options considered lead to 12 specific alternative scenarios for the time horizon of 2016-2026 in addition to the RC (see Table 3). Recognizing that the pre-OPV2 cessation modeling recommended shifting to IPV for oSIAs 5 years after OPV2 cessation (21, 23, 32), combined with the modeled use of IPV in some iterations motivating the recommendation for 6 rounds of oSIAs in very high transmission areas (i.e., in blocks with WPV1 R_0_ > 11) in those studies, we also ran a sensitivity analysis to explore the recommended oSIA characteristics, but always using 4 rounds (i.e., not using 6 rounds for the very high transmission blocks). Although pre-OPV2 cessation modeling also recommended restarting OPV in RI in the event of widespread transmission and cases above a specified threshold (23), the current GPEI strategic plan does not include OPV2 restart as an option (45). Consequently, we run the prospective look back analysis without OPV2 restart. In contrast to pre-OPV2 cessation modeling that focused on quantification of the probability of OPV restart as an outcome, for this analysis with no OPV restart, we focus on the probability of die out of transmission at the end of the time horizon (POD) (49).

**Table 3:** Estimated expected value (median) and [range] of poliovirus cases in 100 stochastic iterations for 2016–2026 for the scenarios modeled.

**Scenario**	***IPV in all RI***		***No IPV prerequisite***	
**Estimated expected global cVDPV2 cases (median) [range]**				
mOPV2, current oSIA	13,981 (9,857)	[1,073–47,975]	62,913 (59,930)	[3,527–141,974]
nOPV2 no reversion, current oSIA	5,297 (2,464)	[433–34,857]	13,256 (7,407)	[766–103,320]
nOPV2 some reversion, current oSIA	15,000 (12,340)	[1,667–60,793]	65,409 (62,659)	[4,417–158,581]
mOPV2, recommended oSIA	354 (102)	[102–4,226]	3,541 (670)	[255–33,378]
nOPV2 no reversion, recommended oSIA	136 (98)	[98–1,525]	369 (254)	[254–2,537]
nOPV2 some reversion, recommended oSIA	295 (103)	[103–4,979]	4,447 (661)	[255–31,885]
**Estimated expected global total** ^*^ **type 2 cases (median) [range]**				
mOPV2, current oSIA	14,285 (10,123)	[1,125–48,948]	64,954 (61,906)	[3,678–143,964]
nOPV2 no reversion, current oSIA	5,396 (2,508)	[488–34,964]	13,441 (7,557)	[872–103,577]
nOPV2 some reversion, current oSIA	15,186 (12,445)	[1,710–61,521]	66,332 (63,722)	[4,513–160,006]
mOPV2, recommended oSIA	432 (136)	[136–4,530]	4,164 (1,117)	[342–34,510]
nOPV2 no reversion, recommended oSIA	188 (129)	[129–1,595]	468 (348)	[306–2,796]
nOPV2 some reversion, recommended oSIA	357 (136)	[136–5,110]	4,782 (841)	[332–32,845]

* Global total cases include all type 2 cases, including vaccine-associated paralytic polio (VAPP) and type 2 vaccine-derived poliovirus (VDPV2) cases that do not meet the laboratory criteria for cVDPV2.

cVDPV2, circulating vaccine-derived poliovirus type 2; mOPV2, monovalent Sabin oral poliovirus vaccine type 2; nOPV2, novel oral poliovirus vaccine type 2; some reversion, some reversion to wild-type phenotype; no reversion, no reversion to wild-type phenotype; oSIA, outbreak response supplemental immunization activity; RI, routine immunizations.

We simulated 100 stochastic iterations starting with the same random number seeds and initial conditions for each scenario. To ensure consistent comparisons for the scenarios from 2017 on, we ignored any reintroductions we previously modeled manually in the deterministic run up for the RC to correspond to actual events that occurred since 2017 (e.g., reintroductions of type 2 transmission in Angola and Pakistan in 2019) (27, 28). Thus, we roll back the clock and allow stochastic events to prospectively occur from 2017 on to explore what the possible futures would have looked like at the end of 2016 with the epidemiological conditions that existed then, but assuming different prospective oSIA strategies. The modeled time horizon of 2016–2026 is bounded by the year of global OPV2 cessation in RI and the end date of the current GPEI strategic plan (45). We note 2021 as a third landmark year in this time horizon, as the most recent full year with epidemiological data. We focus on cVDPV2 incidence, the POD, defined as the number of iterations with no ongoing transmission as of December 31, 2026, as well as the expected number of affected modeled subpopulations out of 720 in the 100 stochastic iterations with a cVDPV2 case for the scenarios modeled. We also report the number of vaccine doses used for the oSIAs by the model, without any adjustment for wastage or inefficiencies in the stockpiling and distribution systems that store, manage, and move vaccines. These dose estimates support direct comparisons between the model scenarios, but not estimates of total vaccine doses that would have been needed. We performed all simulations using JAVA^TM^ programming language in the integrated development environment Eclipse^TM^.

## 4. Results

**Figure 1:**
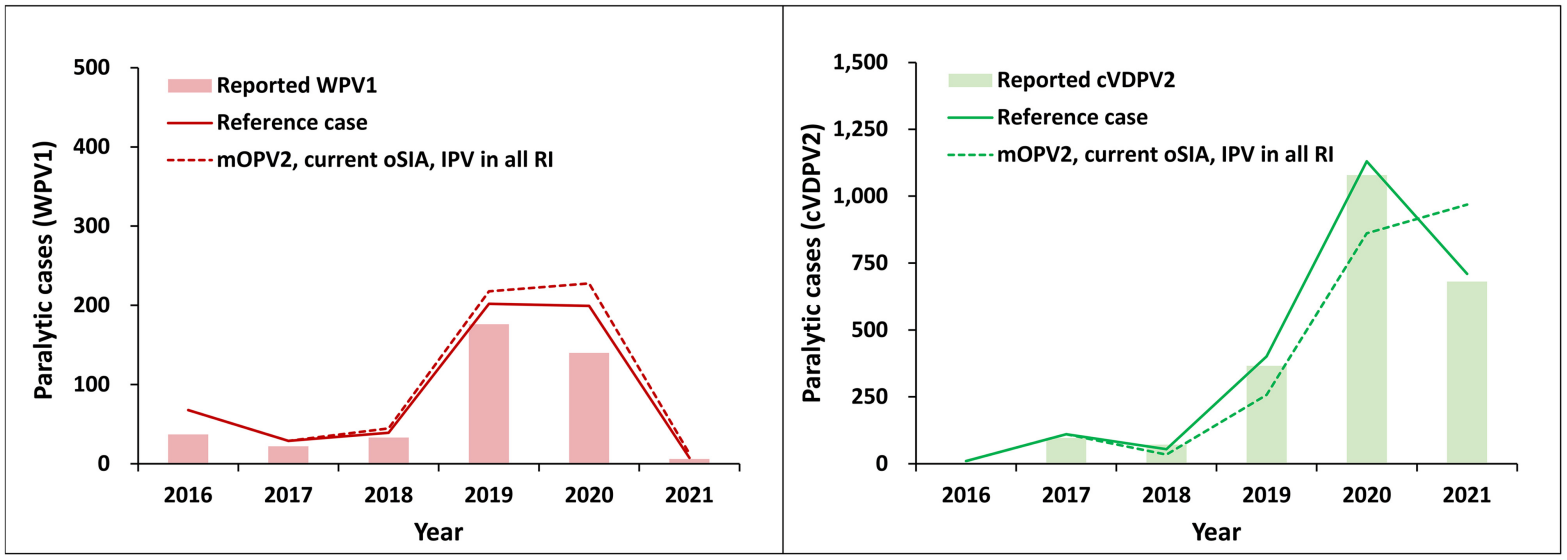
Similarity between the expected global number of paralytic WPV1 **(left panel)** and cVDPV2 **(right panel)** cases by year for 100 stochastic iterations of the “mOPV2, current oSIA” scenario for 2016–2021 (dashed lines) compared to the deterministic reference case (RC, solid lines). In all modeled scenarios, IPV is used in RI in all countries as a prerequisite to OPV cessation. The actual number of reported cases are shown in solid bars for reference.

Figure 1 shows the RC (solid lines), with its deterministic run-up through 2021 (fitted to represent the recent vaccine use and epidemiological history), and the actual reported paralytic cases in solid bars. Results for WPV1 (left panel) and cVDPV2 (right panel) show the deterministic model reference case (RC, solid lines) closely tracks actual data. Figure 1 also shows the expected average of 100 possible futures when looking prospectively from 2016 with uncertainty about stochastic events simulated for 2017 to 2021 (dashed lines). These results, for which the first row of Table 3 provides key summary statistics, do not reflect fitting for actual importation and outbreak events that occurred, but stochastically represent expectations about possible futures associated with the risks, policies, strategies, and implemented performance between 2017 and 2021. Specifically, the comparator scenario to the RC includes IPV use in all RI, mOPV2 for outbreak response, and the *current* outbreak response characteristics, as listed in Table 1.

**Figure 2:**
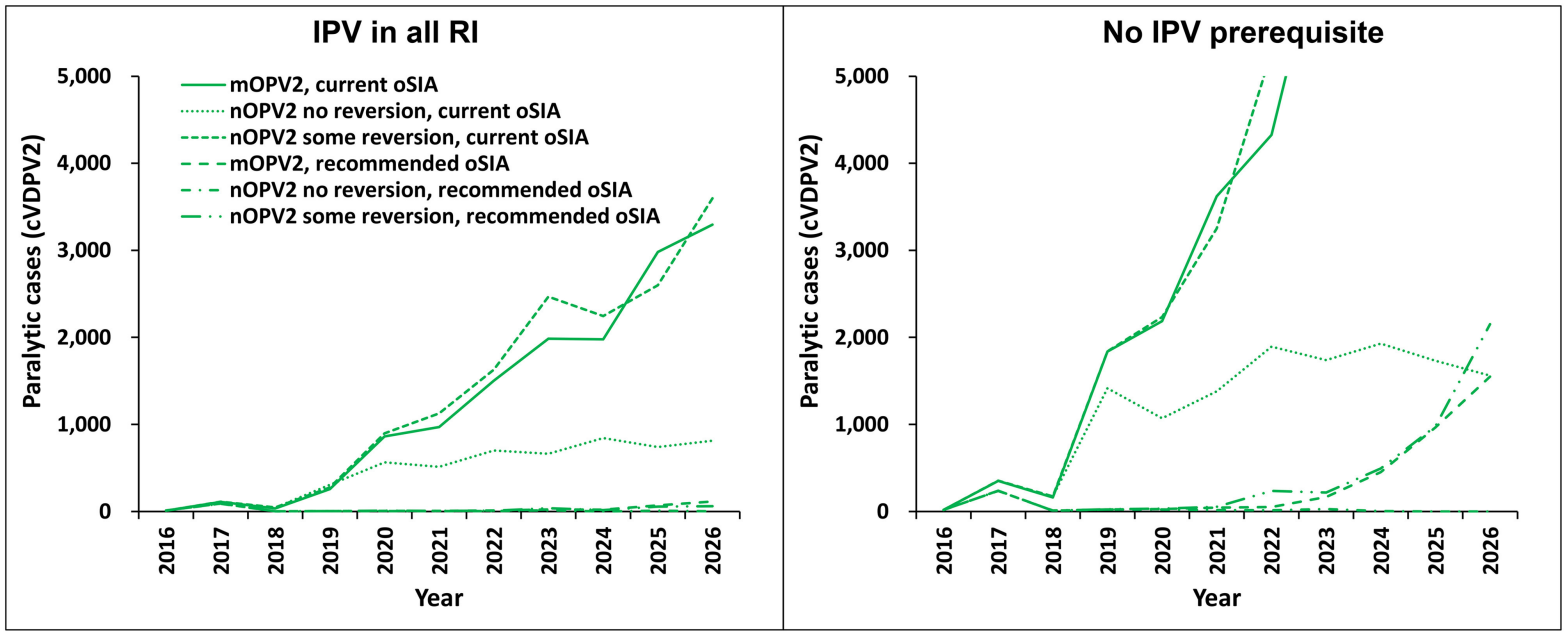
Modeled expected global number of paralytic cVDPV2 cases by year for 100 stochastic iterations of the different vaccine choices and oSIA characteristics for the period 2016–2026. The figure legend shows outbreak vaccine choice and response characteristics. The panel on the **left** shows results for modeled scenarios in which IPV is used in RI in all countries as a prerequisite to OPV cessation. The panel on the **right** shows results for modeled scenarios in which there is no prerequisite for introduction of IPV into RI in countries classified as low-income or lower middle-income by the World Bank.

For the entire time horizon (2016–2026), we focus on the cVDPV2 cases, as these represent an ongoing challenge in global efforts to manage the post-OPV2 cessation paralytic polio cases and risks. For the modeled scenarios presented in Figure 2 and key statistics summarized in Tables 2–4, we use the scenario labeled as “mOPV2, current oSIA” with the prerequisite of IPV in all RI as the comparator that best represents the conditions and policies in the RC.

**Table 4:** Estimated probability of type 2 transmission die-out (defined as the number of iterations with no ongoing transmission as of December 31, 2026) and the expected value (median) [range] of affected modeled subpopulations out of 720 in 100 stochastic iterations for the scenarios modeled.

	**Probability of die out (%)**		**Estimated expected affected modeled subpopulations (out of 720) (median) [range]**	
**Scenario**	**IPV in all RI**	**No IPV prerequisite**	**IPV in all RI**	**No IPV prerequisite**
mOPV2, current oSIA	34	3	41 (34) [6–138]	102 (105) [10–208]
nOPV2 no reversion, current oSIA	64	51	19 (11) [3–114]	26 (18) [5–179]
nOPV2 some reversion, current oSIA	33	6	41 (34) [8–173]	91 (89) [9–221]
mOPV2, recommended oSIA	89	52	5 (3) [3–30]	23 (17) [4–94]
nOPV2 no reversion, recommended oSIA	100	100	3 (3) [3–17]	5 (4) [4–21]
nOPV2 some reversion, recommended oSIA	93	57	4 (3) [3–39]	17 (9) [4–77]

IPV, inactivated poliovirus vaccine; bOPV2, bivalent Sabin oral poliovirus vaccine; mOPV2, monovalent Sabin oral poliovirus vaccine type 2; nOPV2, novel oral poliovirus vaccine type 2; oSIA, outbreak response supplemental immunization activity; RI, routine immunizations.

Figure 2 shows the expected value of annual cVDPV2 paralytic cases as a function of time for two sets of six modeled scenarios. The left panel of curves shows results for scenarios that include IPV in all RI (i.e., all countries) as a policy prerequisite to OPV cessation. The right panel of six curves represents results in which IPV use in RI is not a policy prerequisite, such that the model does not include IPV use in RI in LI and LMI population blocks. Within each panel, three scenarios show the *current* oSIA characteristics (described in Table 1, right column), and three scenarios show the *recommended* oSIA characteristics (described in Table 1, middle column), with the three scenarios showing different vaccines used for oSIAs (i.e., mOPV2, nOPV2 no reversion, and nOPV2 some reversion). Notably, none of the six modeled scenarios that use the “current oSIA” characteristics (Table 1) have a high probability of disease eradication. In contrast, all four scenarios that lead to a high probability of disease eradication require “recommended oSIA” characteristics, including all three scenarios with “recommended oSIA” and prerequisite IPV in RI in all countries (left panel), as well as the “nOPV2 no reversion, recommended oSIA” scenario with no IPV prerequisite in low-income and lower middle-income countries (right panel).

Tables 3, 4 report key summary statistics that complement the results in Figure 2, which provide insights about the range of potential outcomes for each of the modeled scenarios. The ranges highlight the reality that chance events, such as introduction of virus into high transmission populations, can results in significantly larger numbers of paralytic cases than the expected values shown in Figure 2. The top of Table 3 reports only on cVDPV2 cases, and the bottom of Table 3 reports all paralytic cases caused by type 2 polioviruses, which includes VAPP as well as VDPV2 cases that do not meet laboratory criteria as cVDPV2s. Table 4 shows that only the scenarios that used the recommended oSIAs (the bottom 3) show high PODs and relatively low expected spread as measured by the number of affected modeled subpopulations. The scenarios that include IPV in RI as a prerequisite, and particularly the scenario with nOPV2 no reversion, performed best for this analysis.

Overall, our results reveal four important concepts. First, there are no scenarios with “current oSIA” characteristics that result in a POD over 70% by 2026 (top of Table 3). Notably, Figure 2 shows increasing trends for the number of paralytic cases at the end of the time horizon in five of six modeled scenarios with “current oSIA.” Second, the prerequisite of IPV in RI in all countries (Figure 2, left panel) in addition to “recommended oSIA” performance results in POD over 85%, regardless of oSIA vaccine choice. Table 4 shows that an ideally performing nOPV2 (i.e., nOPV2 no reversion) is superior under these circumstances, although this upper bound may prove more optimistic than real-world data on nOPV2 performance as we learn more from its use (53). Third, without IPV in RI (i.e., no IPV prerequisite, Figure 2, right panel), the only scenario with POD over 60% is the ideal “nOPV2 no reversion” vaccine in addition to “recommended oSIA” performance. Finally, the use of IPV in all RI substantially reduces the numbers of expected paralytic cases for all scenarios with “current oSIA” performance characteristics, which suggests that IPV in RI is providing some paralysis insurance benefit for those children who receive it and for which IPV represents their only source of immunity to type 2.

Considering the “mOPV2, recommended oSIA” characteristics under the “IPV in all RI” option without expanding to 6 rounds for the very high transmission blocks, we found that 4 rounds led to similar POD estimates given the prolonged availability of OPV2 for outbreak response (similar results not shown). In a separate sensitivity analysis, the shift to IPV for oSIAs at 5 years after OPV2 cessation, instead of using OPV2 for outbreak response throughout the time horizon made things worse (i.e., substantially decreased the POD and substantially increased the expected incidence, results not shown).

Finally, Table 5 shows that use of the recommended oSIA characteristics substantially reduces the expected vaccine doses required for oSIAs compared to the current oSIA characteristics. In addition, the use of IPV in RI also reduces the expected OPV doses needed for oSIAs. Notably, even with the actual initial conditions that existed in 2016, implementation of the recommended oSIA characteristics using mOPV2 would likely have used less mOPV2 than the doses available in the stockpile created prior to OPV2 cessation. This differs significantly from the situation that occurred, which included restart of new OPV2 bulk production (both Sabin and novel) after OPV2 cessation, but no restart of OPV2 in RI to date.

**Table 5:** Estimated expected value (median) and [range] of vaccine use for outbreak response in 100 stochastic iterations for 2016–2026 for the scenarios modeled.

**Scenario**	**Estimated expected millions of oSIA doses used (median) [range]**							
	**bOPV**		**mOPV2**		**nOPV2**		**IPV**	
**IPV in all RI:**								
mOPV2, current oSIA	46 (13)	[13–139]	576 (518)	[158–1,505]	NA		0.4 (0)	[0–27]
nOPV2 no reversion, current oSIA	40 (13)	[13–190]	NA		358 (278)	[126–1,028]	0.1 (0)	[0–6]
nOPV2 some reversion, current oSIA	46 (13)	[13–138]	NA		585 (561)	[162–1,541]	0.2 (0)	[0–7]
mOPV2, recommended oSIA	55 (54)	[54–123]	284 (128)	[128–1,667]	NA		0.4 (0)	[0–15]
nOPV2 no reversion, recommended oSIA	54 (54)	[54–58]	NA		141 (127)	[127–537]	0.3 (0)	[0–14]
nOPV2 some reversion, recommended oSIA	55 (54)	[54–122]	NA		264 (128)	[128–1,496]	0.4 (0)	[0–14]
**No IPV prerequisite:**								
mOPV2, current oSIA	68 (63)	[13–342]	1,641 (1,619)	[681–2,840]	NA		1.1 (0)	[0–34]
nOPV2 no reversion, current oSIA	72 (96)	[13–197]	NA		490 (413)	[177–1,923]	0.5 (0)	[0–26]
nOPV2 some reversion, current oSIA	82 (90)	[13–342]	NA		1,562 (1,537)	[667–2,996]	0.9 (0)	[0–34]
mOPV2, recommended oSIA	56 (54)	[54–183]	1,856 (1,495)	[317–5,218]	NA		0.5 (0)	[0–15]
nOPV2 no reversion, recommended oSIA	54 (54)	[54]	NA		198 (176)	[176–641]	0.3 (0)	[0–14]
nOPV2 some reversion, recommended oSIA	55 (54)	[54–89]	NA		1,754 (1,409)	[262–4,574]	0.4 (0)	[0–14]

IPV, inactivated poliovirus vaccine; bOPV2, bivalent Sabin oral poliovirus vaccine; mOPV2, monovalent Sabin oral poliovirus vaccine type 2; nOPV2, novel oral poliovirus vaccine type 2; oSIA, outbreak response supplemental immunization activity; RI, routine immunizations.

## 5. Discussion

The results of this and other analyses (49) suggest that the current oSIA strategies appear insufficient to achieve the goals of the 2022–2026 GPEI strategic plan (45), regardless of the vaccine choice. Looking back at the post-OPV2 cessation timeframe, our results show that if countries had performed oSIAs as recommended by pre-OPV2 cessation modeling, then the 2016 globally coordinated OPV2 cessation would have had a high probability of success by 2022. Compared to pre-OPV2 cessation estimates of 94% chance of success (23), using mOPV2 for oSIAs with the updated initial conditions and risks dropped the probability to 87% (Table 4, mOPV2, recommended oSIA for IPV in all). Unfortunately, perceptions about Sabin vaccine risks continue to lead to greater focus on the development and promotion of new vaccines, instead of overcoming the failure to vaccinate (34). If the global community cannot identify ways to improve oSIA performance, then the prospects for successful OPV cessation appear low, and national and global health leaders may need to consider restarting OPV2 in RI (41).

Modeling exercises like this provide an opportunity to learn from the past and guide the future. By rolling back the clock and exploring different prospective futures, we can learn from prior missteps and potentially identify better prospective options. For example, the availability of an ideal vaccine (such as nOPV2 no reversion in our simulations) for immediate use after the 2016 OPV2 cessation could have increased the chances of successful OPV2 cessation with current oSIA performance and increased the POD to as high as 64% if nOPV2 showed equal effectiveness for secondary spread to mOPV2 but did not revert or cause VAPP (Table 4). In addition, if nOPV2 performed that well, then this could have reduced hesitancies to use OPV2 for wider and better oSIAs. The actual performance characteristics of nOPV2 in the field remain under investigation to support eventual licensure (53). Early experience to date suggests that some reversion (i.e., loss of attenuating mutations) may occur and that nOPV2 may prove relatively less effective than mOPV2 when used in populations (46, 49). Nonetheless, optimism about nOPV2 performance may motivate the consideration of requiring the availability of a novel OPV for types 1 and 3 prior to the coordinated cessation of bivalent OPV. This analysis did not consider the trade-offs of delaying OPV2 cessation (beyond 2016) to wait for nOPV2, which would have likely included both additional costs and cases. The GPEI did not consider delaying OPV2 cessation or the option of cessation of both OPV types 2 and 3, despite modeling that explored this option (54) when it committed to implement OPV2 cessation in 2016. The GPEI would not likely have delayed OPV2 cessation to wait for increased supplies of a new vaccine given that it did not delay OPV2 cessation despite not meeting all of its readiness criteria for OPV2 cessation. The experience with OPV2 cessation may change perceptions about the feasibility and the logistics of OPV cessation as a GPEI strategy, which may impact the polio endgame trajectories for types 1 and 3.

Although the difference between the actual epidemiological data, deterministic RC, and expected value of stochastic runs in Figure 1 is small, review of the range of potential outcomes in the stochastic runs (also see first row of Table 3) suggests the potential for significantly higher number of cases for both WPV1 and cVDPV2. Notably, the stochastic runs include some iterations in which simulated outbreaks occur in a high transmission setting, such as India or Bangladesh, although since 2016 no such events have actually occurred to date (5). Since transmission in these countries in our model drives higher expected global burdens of disease, this makes the expected values of the stochastic iterations (dashed lines) slightly higher than the RC (solid lines). As such, our results imply that the world was fortunate to date that no exported viruses reintroduced transmission into those areas as of early 2023 (5). Similar observations of high transmission iterations in stochastic modeling performed prior to OPV2 cessation (23) motivated inclusion of OPV restart into RI upon reaching a threshold number of cases after OPV cessation.

Our analyses provide further health economic insights into the post-OPV cessation era. First, these results provide support for the decision to require IPV in RI in all countries based on the absolute number of paralytic cases (Figure 2 left panel vs. right panel). However, these benefits draw directly from the protection from paralysis for children who received the IPV doses in the context of failing to stop OPV2 transmission. The programmatic focus on IPV introduction potentially affected efforts to perform better mOPV2 oSIAs and/or to develop nOPV2 as an option earlier in the polio endgame, although the extent of these impacts remains unknowable. Second, our results extend and reaffirm the urgent need for full field characterization of nOPV2 given that as of February 25, 2023, more than 585 million doses have been administered in 27 countries (55), and the GPEI identified nOPV2 as the vaccine of choice for outbreak response prior to it receiving emergency use licensure (29).

The results of this analysis depend on a number of assumptions, model limitations, and available information [see details in Appendix of (27, 38)]. Specifically, the limitations include the conceptual characterization of global variability using a block/subpopulation structure and the simplified modeling structures used to simulate effective poliovirus introductions during exportation to a new block/subpopulation, transmission die-out, waning of immunity, OPV evolution and the field characteristics of nOPV2.

As the GPEI partners consider prospective strategies for managing the risks of OPV cessation, including the role of nOPV, this analysis adds further support for improving the performance of immunization activities and suggests the need for focusing on finding solutions to ongoing failures to vaccinate instead of concerns about vaccine failure (34). In the context of limited GPEI resources, these results may prove informative for discussions of future GPEI plans for OPV cessation and potentially restarting the use of OPV in RI. If nOPV1 and nOPV3 can be developed very quickly and perform as well or better than the homotypic Sabin OPV, then this analysis suggests that they may offer a means to reduce risks and cases following bOPV cessation. However, future analyses would need to explore the specific options in the context of the current RC (49).

## Data availability statement

The original contributions presented in the study are included in the article/supplementary material, further inquiries can be directed to the corresponding author.

## Author contributions

KT conceived and designed the study. DK performed the modeling analyses. KT, DK, and KB wrote the paper and participated in revising the paper. All authors contributed to the article and approved the submitted version.
